# Differences in oxylipin profile in psoriasis versus psoriatic arthritis

**DOI:** 10.1186/s13075-021-02575-y

**Published:** 2021-07-24

**Authors:** Roxana Coras, Arthur Kavanaugh, Angela Kluzniak, Dustina Holt, Amy Weilgosz, Armando Aaron, Oswald Quehenberger, Christopher Ritchlin, Monica Guma

**Affiliations:** 1 Department of Medicine, School of Medicine, University of California San Diego, 9500 Gilman Drive, La Jolla, CA 92093 USA; 2 Autonomous University of Barcelona, Campus de la UAB, Plaça Cívica, 08193 Bellaterra, Barcelona, Spain; 3 Department of Medicine, University of Rochester, Medical Center, 601 Elmwood Ave, Rochester, NY 14642 USA; 4 Department of Pharmacology, School of Medicine, University of California San Diego, 9500 Gilman Drive, La Jolla, CA 92093 USA

**Keywords:** Psoriatic arthritis, Psoriasis, Enthesitis, Oxylipins, Skin disease

## Abstract

**Background:**

Oxylipins are biological lipids that have been implicated in inflammation. We previously found that certain oxylipins correlated with clinical manifestations in psoriatic arthritis (PsA) patients. Here, we compare oxylipin profiles in PsA patients and those with psoriasis (PsO) without inflammatory arthritis to identify oxylipins that associate with specific disease manifestations to better understand disease pathogenesis and identify new biomarkers.

**Methods:**

Consecutive patients with PsA (who met the CASPAR classification criteria for PsA) and PsO were recruited from the Rheumatology Outpatient Clinic. A thorough clinical examination was performed, including entheseal (Leeds enthesitis index (LEI)) and joint involvement (SJC/TJC 66/68). Patients were evaluated for pain and global disease activity on a visual analog scale (VAS) ranging from 0 to 100. This was followed by disease activity scores calculation: cDAPSA (Disease Activity Index for Psoriatic Arthritis) and Psoriasis Area and Severity Index (PASI). Serum oxylipins were determined by mass spectrometry and their association with clinical characteristics (PASI/LEI and cDAPSA) was analyzed using Metaboanalyst 4.0 and R version 3.6.1.

**Results:**

Twenty PsO (average age 52 [10.8], 55% males) and 19 PsA patients (average age 60.5 [11.4], 63.1% males) were included. PsO patients had an average body mass index (BMI) of 33.7 (6.84) and an average PASI of 3.8 (4.2). PsA patients had an average BMI of 31.9 (5.6), TJC of 9.3 (10.41), SJC of 3.7 (4.23), with an average cDAPSA of 23.3 (11.4). 63.1% of PsA patients had enthesitis (average LEI 2.2 [3]) and the same percentage had psoriasis (average PASI 3(5]). Sera were analyzed for oxylipin levels. PsO and PsA patients with higher PASI score (> 2.5) had significantly lower serum concentrations of pro-inflammatory oxylipins, most of them arachidonic acid derived (AA). Oxylipin profiling did not associate with cDAPSA. Interestingly, several AA-derived oxylipins (5,15 di-HETE (5S,15S-dihydroxy-6E,8Z,10Z,13E-eicosatetraenoic acid), 5-oxoETE (5-Oxo-eicosatetraenoic acid), PGE2 (prostaglandin E2), 11bPGE2 (11 beta prostaglandin D2), and LTB4 (leukotriene B4)) were significantly increased in PsA patients with enthesitis compared to those without.

**Conclusions:**

The AA-derived proinflammatory oxylipins were lower in both PsO and PsA patients with higher skin scores. Joint disease activity was not associated with the concentrations of oxylipins. Yet, enthesitis was associated with an increase of AA-derived pro-inflammatory oxylipins in PsA patients. Further studies are needed to determine whether oxylipin profiling can be a good biomarker of enthesitis in PsA patients.

**Supplementary Information:**

The online version contains supplementary material available at 10.1186/s13075-021-02575-y.

## Introduction

Psoriatic arthritis (PsA) is a systemic inflammatory disease that involves, in addition to axial and peripheral arthritis, extraarticular manifestations, including nail changes, dactylitis, and enthesitis [1]. The etiology and pathogenesis of PsA is not fully understood but involves a complex interaction between genetic and environmental factors resulting in immune-mediated inflammation of the skin and joints [2]. The risk factors that predispose patients to develop PsA, psoriasis (PsO), and other extraarticular manifestations do not always overlap, and joint, skin, and enthesis do not respond to the same extent to some of the current therapies, suggesting an underlying heterogeneity of its physiopathology [3].

Oxylipins are bioactive lipids related to several processes including inflammation. In recent years, the development of mass spectrometry techniques has allowed the identification of a high number of oxylipins, compared to the few that are classically known to be involved in inflammation, such as prostaglandins, thromboxanes, and leukotrienes. Oxylipins are synthesized from fatty acids with the intervention of cyclooxygenases (COX), lipoxygenases (LOX5, 12, and 15), cytochrome P450, or non-enzymatically. Omega-6 polyunsaturated fatty acids (PUFA): arachidonic acid (AA), linoleic acid (LA), and dihomo-gamma linolenic acid (DGLA), as well as omega 3 PUFAs: eicosapentaenoic acid (EPA) and docosahexaenoic acid (DHA), are the main precursors of oxylipins (see Supplementary Figure 1). AA and LA are considered to be precursors of mostly pro-inflammatory oxylipins, while EPA, DHA, and DGLA are considered precursors of anti-inflammatory oxylipins and specialized pro-resolving mediators [4].

Since oxylipins control many physiological and pathological processes and they are upstream of inflammatory activity, we hypothesized that they could be implicated in the pathogenesis of the psoriatic disease. In a previous publication from our group [5], we described several AA-derived pro-inflammatory oxylipins and EPA-derived anti-inflammatory oxylipins which correlated with joint disease, suggesting that an imbalance between pro- and anti-inflammatory oxylipins could be involved in the pathogenesis of PsA. However, the heterogeneity in the extraarticular manifestations in that cohort of patients with PsA did not allow us to further understand the differences in the oxylipin profiling in this disease. Here, we compare the oxylipin profile in two different cohorts, one cohort of patients with PsO without arthritis and another cohort of patients with PsA, to study differences in oxylipins in both cohorts as well as oxylipins associated to specific disease manifestations. The identified oxylipins could help not only to better understand disease pathogenesis, but also be biomarkers for each of the clinical manifestations.

## Patients and methods

This is a cross-sectional study that enrolled patients with PsO without joint involvement, and patients with PsA who fulfilled the classification for PsA (CASPAR) criteria [6]. Consecutive patients were recruited from the Rheumatology Outpatient Clinic of the University of Rochester Medical Center. The study was approved by the Institutional Board Review and patients signed an informed consent. A thorough clinical examination was performed, which included a count of the number of tender (TJC out of 68) and swollen joints (SJC out of 66), number of tender entheseal sites (Leeds enthesitis index; range 0–6), and presence/absence of dactylitis, as well as the percentage of body surface that was affected by PsO and calculation of the PASI (Psoriasis Area and Severity Index). Pain and global disease activity were also evaluated by the patient, using a visual analog scale (VAS) that ranged from 0 to 100. The cDAPSA (clinical Disease Activity in PSoriatic Arthritis) score was calculated [7]. Non-fasting blood samples were collected in the clinic by research personnel into 10 ml BD Vacutainer Blood Collection Tubes containing spray-coated silica and a polymer gel for serum separation. After 30 min incubation at room temperature, tubes were centrifuged for 10 min at 2000×*g* and sera were transferred into 1.7 ml tubes and immediately frozen and stored at − 80 °C until analysis.

### Lipid extraction and LC-MS measure of oxylipins

All sera samples at baseline were stored at − 80 °C, thawed once, and immediately used for free fatty acid and oxylipin isolation as previously described [8]. Briefly, 50 μL sera was spiked with a cocktail of 26 deuterated internal standards that also included some selected PUFAs (individually purchased from Cayman Chemicals, Ann Arbor, MI) and brought to a volume of 1 mL with 10% methanol. The samples were then purified by solid-phase extraction on Strata-X columns (Phenomenex, Torrance, CA), using an activation procedure consisting of consecutive washes with 3 mL of 100% methanol followed by 3 mL of water. The oxylipins were then eluted with 1 mL of 100% methanol, which was dried under a vacuum, dissolved in 50 μL of buffer A (consisting of water–acetonitrile–acetic acid, 60:40:0.02 [v/v/v]), and immediately used for analysis. Oxylipins in sera were analyzed and quantified by LC/MS/MS as previously described [8, 9]. Briefly, oxylipins were separated by reverse-phase chromatography using a 1.7 μm 2.1 × 100 mm BEH Shield Column (Waters, Milford, MA) and an Acquity UPLC system (Waters). The column was equilibrated with buffer A, and 10 μL of sample was injected via the autosampler. Samples were eluted with a step gradient starting with 100% buffer A for 1 min, then to 50% buffer B (consisting of 50% acetonitrile, 50% isopropanol, and 0.02% acetic acid) over a period of 3 min, and then to 100% buffer B over a period of 1 min. The LC was interfaced with an IonDrive Turbo V ion source, and mass spectral analysis was performed on a triple quadrupole AB SCIEX 6500 QTrap mass spectrometer (AB SCIEX, Framingham, MA). Oxylipins were measured using electrospray ionization in negative ion mode and multiple reaction monitoring (MRM) using the most abundant and specific precursor ion/product ion transitions to build an acquisition method capable of detecting 158 analytes and 26 internal standards. The ionspray voltage was set at − 4500 V at a temperature of 550 °C. Collisional activation of the oxylipin precursor ions was achieved with nitrogen as the collision gas with the declustering potential, entrance potential, and collision energy optimized for each metabolite. Oxylipins were identified by matching their MRM signal and chromatographic retention time with those of pure identical standards.

Oxylipins were quantitated by the stable isotope dilution method. Briefly, identical amounts of deuterated internal standards were added to each sample and to all the primary standards used to generate standard curves. To calculate the amount of oxylipins and free fatty acids in a sample, ratios of peak areas between endogenous metabolites and matching deuterated internal standards were calculated. Ratios were converted to absolute amounts by linear regression analysis of standard curves generated under identical conditions. Oxylipin levels are expressed in picomol/milliliter (pmol/mL). To account for batch effects, quality control samples were run in each batch; the average coefficient of variance for the quantified oxylipins was 4% (standard deviation 0.01).

The oxylipins identified and quantified in our study are derived from the fatty acid precursors: omega-6 polyunsaturated fatty acids (PUFA) - arachidonic acid (AA), linoleic acid (LA), and dihomo-gamma linolenic acid (DGLA), as well as omega 3 PUFAs: eicosapentaenoic acid (EPA) and docosahexaenoic acid (DHA) (see Supplementary Figure 1)

### Data analysis

Numerical variables were expressed as mean (standard deviation) and categorical variables as percentage. Partial Least Square Regression Discriminant Analysis (PLS-DA) was used to select features that discriminated between groups with previous quantile normalization and autoscaling of the variables. The top 15 selected variables according to the VIP (variable importance in projection) score were then used to create a heatmap to visualize oxylipin patterns according to the selected groups. Clustering was performed using the Euclidean distance and a ward clustering algorithm. Spearman correlation was performed between clinical variables (PASI, TJC, SJC, VAS activity, VAS Pain, and cDAPSA) and each oxylipin. The correlation coefficients and the *p* values were used to build heatmaps. Non-parametric tests were used to compare the means of different groups. Metaboanalyst 4.0 (https://www.metaboanalyst.ca/MetaboAnalyst/ModuleView.xhtml) and R version 3.6.1 were used to perform statistical analysis.

## Results

### Patient demographics and disease characteristics

Nineteen PsA and 20 PsO patients were recruited. The demographics of the patients, along with the disease characteristics and treatment they received are summarized in Tables 1 and 2. The average age of PsA patients was 60.5 years old (± 11.4) where 63.1% were males, with an average body mass index (BMI) of 31.9 (± 5.6). The average number of tender joints (TJC) was 9.3 (± 10.4) and of swollen joints (SJC) 3.7 (± 4.2), with an average of 59.1 (± 28.7) VAS activity. The patients had moderate disease activity with an average cDAPSA of 23.4 (±11.4). 63.1% of patients had enthesitis, with an average score of 2.2 (± 3), and the same percentage also had mild psoriasis (PASI < 12 and BSA < 10), with an average PASI of 3 (± 5.1) and average BSA of 4.7(± 11). PsO patients’ average age was 51.9 (± 10.8) and 55% were males, with an average BMI of 33.7 (± 6.8). They had mild psoriasis, with an average PASI of 3.8 (± 4.2) and an average BSA of 4.5 (± 4.7). None of the PsO patients was receiving TNF alpha inhibitors or systemic corticosteroids, while 75% were receiving topical corticosteroids and 10% were receiving a DMARD. None of the PsA patients was receiving systemic corticosteroids, while 47.3% were receiving TNF-alpha inhibitors and 26.3% were on a DMARD.

**Table 1:** Demographic and disease characteristics for PsA patients

Variable	Psoriatic arthritis (*N*=19)
Age (years) (mean, SD)	60.5 (± 11.4)
Sex F:M	7:12 (63.1% male)
BMI (kg/m^2^) (mean, SD)	31.9 (± 5.6)
PsO (%)	63.1%
PASI	3 (± 5.1)
BSA (%)	4.7 (± 11)
TJC (mean, SD)	9.3 (± 10.4)
SJC (mean, SD)	3.7 (4.2)
VAS Activity (mean, SD)	59.1 (± 28.7)
VAS Pain (mean, SD)	43.5 (± 32.2)
Enthesitis (%)	63.1%
Enthesitis (mean, SD)	2.2 (± 3)
cDAPSA	23.4 (11.4)
NSAIDs (%)	16.7%
DMARDs (%)	26.3%
TNF alpha inhibitors (%)	47.3%
Corticosteroids (%)	0
Topical corticosteroids (%)	26.3%

*BMI* body mass index, *PASI* Psoriasis Area and Severity Index, *BSA* body surface area, *TJC* tender joint count, *SJC* swollen joint count, *VAS* visual analog scale, *HAQ* Health Assessment Questionnaire, *NSAID* non-steroid anti-inflammatory drugs, *TNF* tumor necrosis factor, *DMARDs* disease-modifying anti-rheumatic drugs.

**Table 2:** Demographic and disease cCharacteristics for PsO patients

Variable	Psoriasis group *N* = 20
Age (years) (mean, SD)	51.9 (± 10.8)
Sex F:M	9:11 (55% male)
BMI (kg/m^2^) (mean, SD)	33.7 (± 6.8)
PASI (mean, SD)	3.8 (± 4.2)
BSA (mean, SD)	4.5 (± 4.7)
TJC	3.05 (4.8)
Enthesitis (%)	25%
Enthesitis (LEI mean)	1.5 (2.79)
NSAIDs (%)	35%
DMARDs	10%
TNF alpha inhibitors (%)	0
Corticosteroids (%)	0
Topical corticosteroids (%)	75%

*BMI* body mass index, *PASI* Psoriasis Area and Severity Index, *BSA* body surface area, *NSAID* non-steroid anti-inflammatory drugs, *DMARDs* disease-modifying anti-rheumatic drugs, *TNF* tumor necrosis factor

### Oxylipin profile depends on the skin disease activity (PASI) in PsO patients

Levels of serum oxylipins were measured in 20 PsO patients. A total of 66 oxylipins derived from arachidonic acid (AA) *(n*=32), docosahexanoic acid (DHA) (*n*=11), linoleic acid (LA) (*n*=6), eicosapentanoic acid (EPA) (*n*=9), dihomo-g-linolenic acid (DHGLA) (*n*=5), and a-linolenic acid (ALA) (*n*=3) were identified by LC/MS in our cohort. No associations with age, sex or BMI were observed (*p* > 0.050, except for 14,15-diHETrE with age, *p*=0.02, and 8,9-diHETrE with sex, *p*=0.03).

**Fig. 1:**
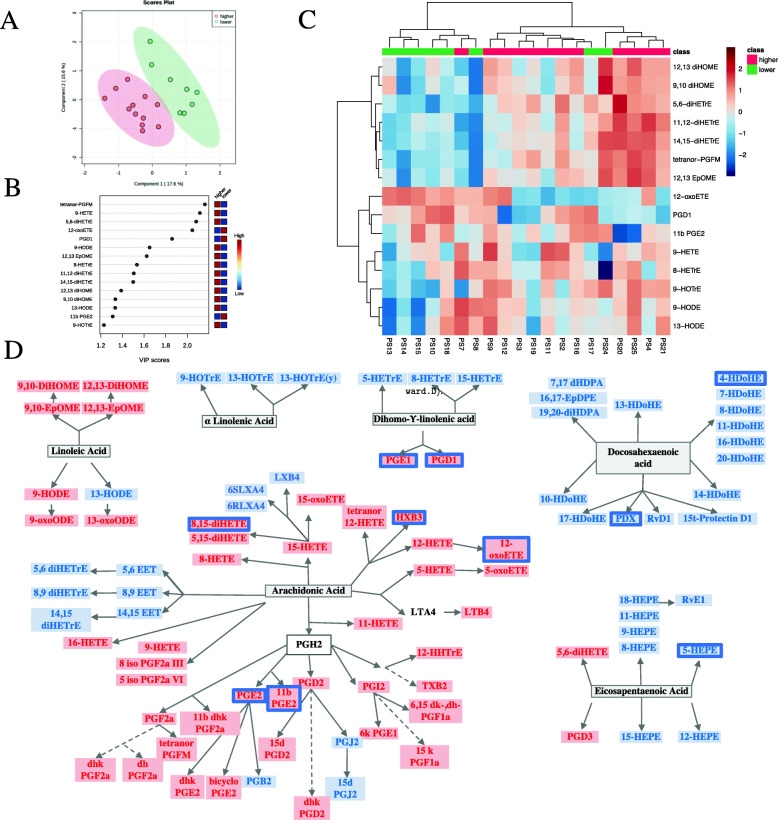
Oxylipin profile is different in PsO patients by skin disease activity. **A** PLS-DA scores plot of PsO patients with higher (*N* = 12) versus lower PASI (*N* = 8). The explained variances for each component are shown in brackets. **B** Important features identified by PLS-DA. The colored boxes on the right indicate the relative concentrations of the corresponding metabolite in each group under study. **C** Heatmap performed using the important features identified by PLS-DA which shows different patterns in the 2 groups. **D** Precursor assignment for the oxylipins that are decreased (blue rectangle) in PsO patients with higher disease activity compared to the lower group

According to skin disease activity evaluated by PASI, PsO patients were classified in 2 groups: low (PASI ≤ 2.5) and moderate skin disease activity (PASI > 2.5). The characteristics of these patients are depicted in Supplementary Table 1. As per PLS-DA, the oxylipin profile was different between the 2 groups (Fig. 1 A) and we identified 15 oxylipins that best discriminated between patients with higher PASI score (> 2.5) and patients with lower PASI score (Fig. 1 B). Figure 1 C shows that the use of the selected oxylipins allows the discrimination of the 2 clusters, based on PASI. Interestingly, levels of both pro- and anti-inflammatory oxylipins were lower in the high PASI group. We further performed classic statistical analysis of the 2 groups. Most of the oxylipins were decreased in the high PASI group (Fig. 1 D and Supplementary Table 2), and were derived from arachidonic acid (AA), via the cyclooxygenase 2 (PGE2, PGF2a, 11bPGE2) and 12- (HXB3) and 15-LOX (8,15-diHETE, 15-oxoETE). In addition, PGD1 and PGD3, derived from DGLA and EPA, respectively, via COX, the anti-inflammatory 11-HEPE derived from EPA, and 4HDoHE derived from DHA, were also decreased in PsO patients with higher skin inflammation (Fig. 1 D). Correlations between oxylipins and PASI scores are showed in Supplementary Table 3.

### Oxylipin profile in PsA patients varies according to skin disease activity but not articular activity

Levels of serum oxylipins were also measured in 19 PsA patients. A total of 64 oxylipins derived from arachidonic acid (AA) (*n*=32), docosahexanoic acid (DHA) (*n*=11), linoleic acid (LA) (*n*=6), eicosapentanoic acid (EPA) (*n*=9), dihomo-g-linolenic acid (DHGLA) (*n*=5), and a-linolenic acid (ALA) (*n*=4) were identified by LC/MS in our cohort. No associations with age or sex were observed (*p* > 0.050, except for iso PGF2a III with sex, *p*=0.04). Of interest, several oxylipins correlated with BMI (Supplementary Table 4).

**Fig. 2:**
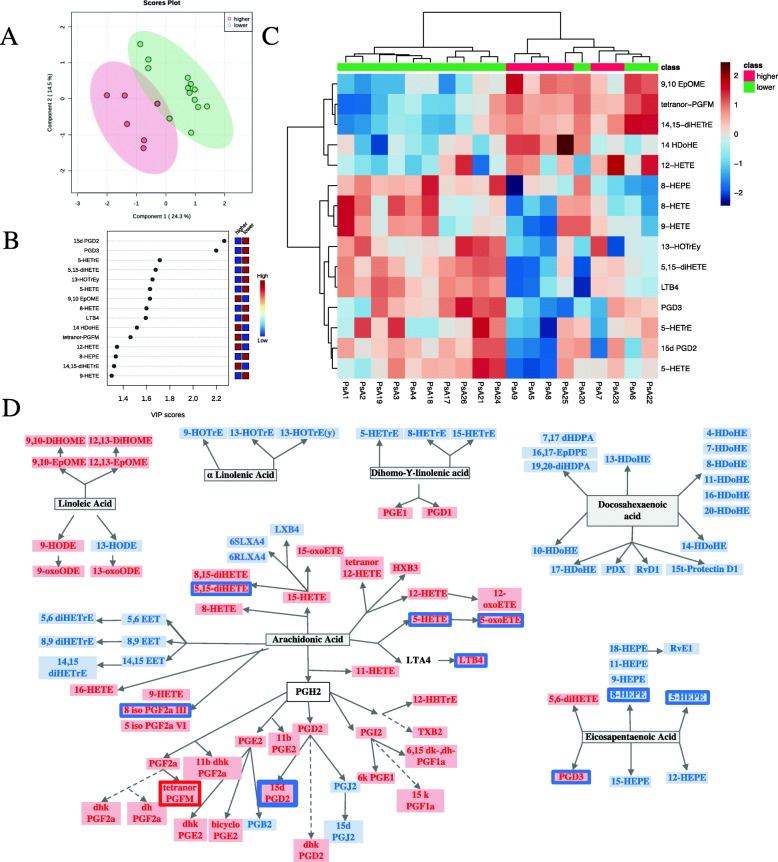
Oxylipin profile is different in PsA patients by skin disease activity. **A** PLS-DA scores plot of PsA patients with higher (*N* = 6) versus lower PASI (*N* = 13). The explained variances for each component are shown in brackets. **B** Important features identified by PLS-DA. The colored boxes on the right indicate the relative concentrations of the corresponding metabolite in each group under study. **C** Heatmap performed using the important features identified by PLS-DA which shows different patterns in the 2 groups. **D** Precursor assignment for the oxylipins that are decreased (blue rectangle) in PsA patients with higher disease activity compared to the lower group. Oxylipins that are increased in PsA patients with higher disease activity are marked in a red rectangle

We also classified PsA patients in 2 groups according to PASI: low skin disease activity (PASI ≤ 2.5) and higher skin activity (PASI > 2.5). The characteristics of the 2 groups are depicted in Supplementary Table 5. As per PLS-DA, the oxylipin profile was different between the 2 groups (Fig. 2 A) and we identified 15 oxylipins that best discriminated between PsA patients with low skin disease activity (PASI ≤ 2.5) and higher skin activity (PASI> 2.5) (Fig. 2 B). Figure 2 C shows how the use of the selected oxylipins allows the discrimination of the groups, based on PASI (high versus low). We further performed classic statistical analysis of the 2 groups. Similar to what we observed in the PsO cohort, levels of both pro- and anti-inflammatory significant oxylipins were lower in PsA patients with higher PASI. This can be seen in the AA-derived pro-inflammatory oxylipins LTB4, 5-HETE, and 5-oxoETE (via 5LOX), as well as lower levels of the anti-inflammatory EPA derived 5- and 8-HEPE, DGLA derived (5-HETrE and 8-HETrE), and DHA derived (17 HDoHE) (Fig. 2 D). 5 iso PGF2a, a non-enzymatic product of AA, is also decreased, while tetranor PGFM, AA derived via COX2, was increased in patients with higher PASI. Comparison of the levels of oxylipins between groups and correlations between oxylipins and PASI scores are showed in Supplementary Tables 6 and 7.

Lastly, we analyzed the association of PASI and nail disease with oxylipins in both cohorts combined (39 patients). Supplementary Table 8 shows that most of the oxylipins are downregulated in patients with PASI more than 2.5 when both PsO and PsA cohorts were combined. However, we did not find any oxylipin associated with mNAPSI (Supplementary Table 9).

**Fig. 3:**
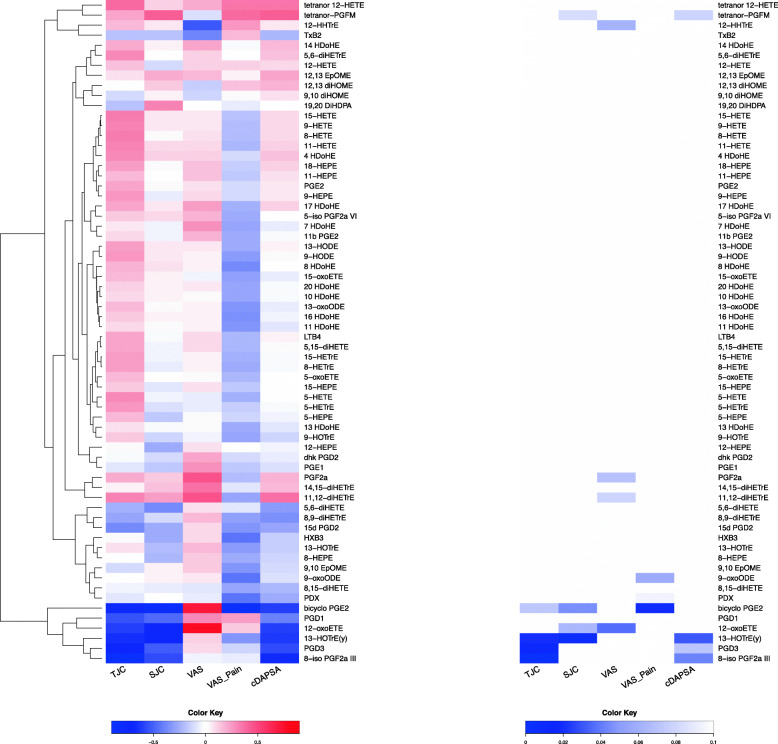
Correlation between serum oxylipins and clinical parameters in PsA patients. **A** Heatmap of the correlation coefficients (Spearman) between each clinical variable and each oxylipin. **B** Heatmap of p values corresponding to the correlations in A

We also evaluated whether the oxylipin profile was associated with articular outcomes. Yet, only a few oxylipins in the PsA patients were different based on the cDAPSA score (Supplementary Tables 10 and 11). Correlation of the oxylipins with the articular outcomes in PsA patients (TJC, SJC, PASI and VAS-Activity, VAS Pain, and cDAPSA) also showed only a few oxylipins that were significantly associated to these outcomes (Fig. 3). The oxylipins that negatively correlated with TJC, SJC, and cDAPSA were bicyclo PGE2, 12-oxoETE, 13-HOTrE, 8 iso PGF2a III, and PGD3.

### Oxylipin profile in PsA patients varies according to enthesitis severity

**Fig. 4:**
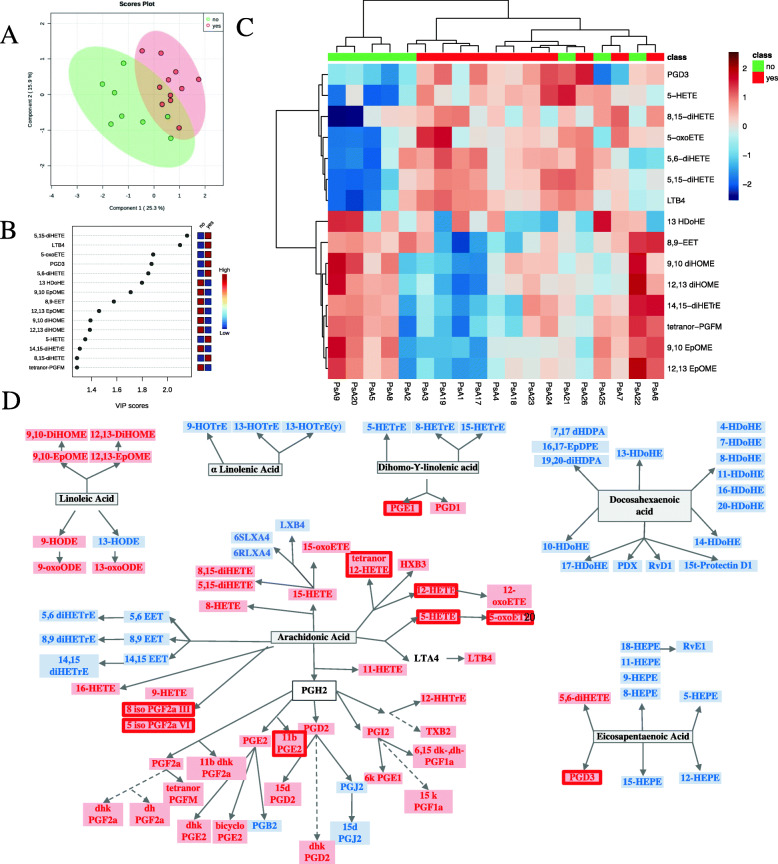
Oxylipin profile is different in PsA patients by enthesis involvement. **A** PLS-DA scores plot of PsA patients with enthesitis (*N* = 11) versus PsA patients without enthesitis (*N* = 8). The explained variances for each component are shown in brackets. **B** Important features identified by PLS-DA. The colored boxes on the right indicate the relative concentrations of the corresponding metabolite in each group under study. **C** Heatmap performed using the important features identified by PLS-DA which shows different patterns in the 2 groups. **D** Precursor assignment for the oxylipins that are decreased (blue rectangle) in PsA patients with enthesitis compared to the group without enthesitis; the oxylipins that are increased in PsA patients with enthesitis compared to the group without enthesitis are indicated by a red rectangle

We then compared PsA patients with enthesitis (enthesitis score > 1) to PsA patients without enthesitis (enthesitis score ≤ 1). The characteristics of the 2 groups are depicted in Supplementary Table 12. Of interest, PsA patients with enthesitis tended to have a higher BMI (34.3 ± 5.6) compared to PsA patients without enthesitis (28.9 ± 4.2, *p* = 0.05), tended to be younger (55.7 ± 10.8 years versus 66.5 ± 9.4 years, *p* = 0.06), and also a higher percentage were women (54.5% versus 12.5, *p* = 0.01). In addition, we used PLS-DA to identify oxylipins which best discriminated between the 2 groups of patients (Fig. 4 A, B). Comparison of the levels of oxylipins between groups is showed in Supplementary Table 13. PGE2, 11bPGE2, 5-oxoETE, and LTB4 were found to be increased in the higher enthesitis PsA group (Fig. 4 C, D and Supplementary Table 13). Supplementary Tables 14 and 15 show the lack of association of enthesitis with oxylipins in PsO and when both cohorts were combined, and the characteristics of PsO patients with more and less enthesitis are presented in Supplementary Table 16.

### There is an overlap of the oxylipin profile in PsO and PsA patients

**Fig. 5:**
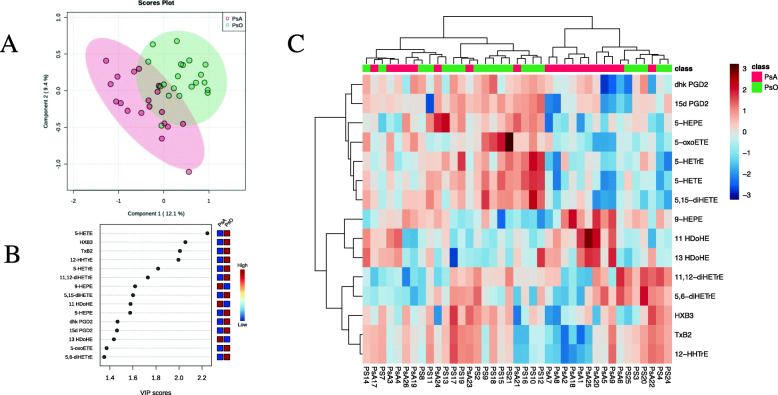
Oxylipin profile in PsA and PsO patients. **A** PLS-DA scores plot of PsA patients (*N* = 19) and PsO patients (*N* = 20). The explained variances for each component are shown in brackets. **B** Important features identified by PLS-DA. The colored boxes on the right indicate the relative concentrations of the corresponding metabolite in each group under study. **C** Heatmap performed using the important features identified by PLS-DA

Supplementary Figure 2 illustrates the overall correlations of the oxylipins in PsA and PsO patients. It can be observed that the 2 diseases present different patterns of clustering of the oxylipins. However, when we used PLS-DA to identify features that discriminate between PsO and PsA patients (Fig. 5 A), we observed an overlap in the oxylipin profiles. Comparisons of the levels of oxylipins between groups are showed in Supplementary Table 17 and do not show any significantly different oxylipin between both groups.

## Discussion

Mechanistic studies are needed to identify key pathobiological factors with a putative role in relation to psoriatic disease and therapeutic response, as well as understand mechanisms driving the different manifestations of this complex disease. Oxylipins and related bioactive lipids constitute an important network and there are previous studies suggesting a role in psoriatic disease pathogenesis. The involvement of oxylipins in the pathogenesis of psoriatic disease has been studied mainly in PsO. Skin lipidomics studies found higher concentrations of AA-derived 8- and 12-HETE and LTB4 in lesional skin compared to non-lesional skin and skin from healthy controls [10–12]. The existence of a natural skin COX inhibitor and a shift towards a LOX-mediated metabolism of AA was suggested to play a role in PsO pathogenesis [12, 13]. Using NMR (nuclear magnetic resonance) spectroscopy, a different lipid profile was described in PsA compared to rheumatoid arthritis (RA) patients [14]. Yet, a lipidomic approach in psoriatic disease to investigate the components of eicosanoid biology, beyond arachidonic acid metabolites and leukotrienes, has not been used before, except for our previous description of the oxylipin profile in patients with PsA [5].

In our study, we evaluated serum oxylipin profiles in patients with PsA or PsO. Patients with PsO had a moderate disease activity and we observed that oxylipins varied according to the severity of skin disease evaluated by PASI. Patients with more severe disease (PASI > 2.5) had a decrease in several serum oxylipins. PGE2 was the oxylipin that best discriminated between the 2 groups. PGE2 is the most abundant prostaglandin in the skin and has a role in the pathogenesis of PsO, since it was shown to be important in the IL (interleukin)-23-dependent generation of pathogenic Th (helper) 17 cells, which are related to PsO pathogenesis [15, 16]. However, it is also involved in the activation of the resolution of inflammation, by stimulating the secretion of specialized pro-resolving mediators [17]. PGE2 and 12- and 15-HETE were described to be higher in psoriatic skin compared to skin from healthy controls, in contrast to serum concentrations that were found to be lower in PsO patients compared to healthy controls [13, 18]. HXB3 was also described to be higher in lesional skin compared to skin from healthy controls [12]. The same studies also found that the concentrations of EPA (5-, 12-, 15-, and 18-HEPE), LA (13-HODE, 9-oxoODE, 13-oxoODE), and DHA (4-, 7-, 14-, and 17-HDoHE) derived oxylipins were higher in lesional skin compared to normal skin, but lower in serum of PsO patients versus healthy controls. Hence, our findings are in agreement with previous studies. Of interest, we reanalyzed the results from our previous PsA cohort [5], and patients with a BSA > 3 (equivalent to higher skin inflammation) also had lower levels of several pro-inflammatory oxylipins in serum (Supplementary Figure 3). Overall, several serum oxylipins derived from all the precursors via COX, 5-, 12-, and 15 LOX seem to be lower in patients with higher skin activity.

The anti-inflammatory oxylipins such as 5- and 8-HEPE (from EPA) and 17HDoHE (from DHA) may decrease in an effort to resolve the skin inflammation in patients with higher skin disease activity. Among the pro-inflammatory oxylipins, local consumption to amplify the inflammatory response could explain the decrease in the release of these oxylipins to the serum. Of interest, receptors for oxylipins are found in the skin and have been described to be involved in the pathogenesis of psoriasis. Using an animal model of skin psoriasis, Ueharaguchi et al observed that TXA2 (AA derived via COX2 and precursor of TXB2) signaling through its receptor, TBXR2, may facilitate psoriatic dermatitis by promoting IL-17 production in psoriatic lesions [19]. Furthermore, mRNA expression of *TBXAR2* was significantly increased in skin biopsies from psoriatic lesions compared to skin from healthy controls [19]. While IL23 induces the production of PGE2 by Th17 cells, PGE2 acts back on its receptors, EP2 and EP4 on these cells, to increase *Il23r* expression in a positive feedback manner. Interestingly, EP4 receptor (*PTGER4*) was overexpressed in human psoriatic lesional skins [15]. These data suggest that, in spite of increased levels of oxylipins in the psoriatic skin, they are probably consumed locally, due to the higher expression of their receptors.

To our surprise, the oxylipin levels did not correlate with joint activity. Yet, oxylipins are also involved in joint inflammation. Data from RA shows that 5LOX and COX2 enzymes are upregulated in the synovium and they remain elevated despite treatment with disease-modifying drugs [20–22]. In addition, 5LOX-deficient mice developed less arthritis in a mouse model of RA [23]. PGE2’s involvement in RA has been also studied. PGE2 receptors (EP1 to 4) are also expressed in the synovium; however, only EP4 was found to be involved in arthritis development in a mouse model of collagen antibody-induced arthritis [24]. Interestingly, polymorphisms of the human EP4 receptor gene *PTGER4* have been identified as risk alleles for ankylosing spondylitis (AS), and its expression by Th17 cells is associated with higher disease activity in AS, but not RA and PsA [25]. PGE2 induces IL17 secretion upon Th17-fibroblast interaction, [26], and this is responsible for worsening arthritis severity in the collagen-induced arthritis model [27]. The lack of a significant association of the detected oxylipins with joint activity in our study could be due to the overlap with skin and entheseal inflammation, yet specific information on eicosanoids and their receptors in PsA synovial tissue is also lacking.

Of interest, we also showed that patients with PsA with a higher enthesitis score had an increase of several oxylipins in serum, some of which were decreased in patients with PsO (PGE2 and 11bPGE2) or PsA (LTB4 and 5-oxoETE) and high PASI. Despite a better understanding of the anatomy and biology of the enthesis, data on the complex mechanisms of enthesis inflammation are lacking. There is limited data on eicosanoids in the enthesis. It was shown that IL17 is produced in the enthesis by γδ T cells and group 3 innate lymphoid cells, and by stimulating PGE2 and IL8 production facilitates neutrophil recruitment and enthesis inflammation. Moreover, expression of COX2 by resident mesenchymal cells reinforces the involvement of PGE2 in the entheseal inflammation [28]. Although more information on the role of eicosanoids in enthesis inflammation is required, our results suggest that specific oxylipins might not only play a role in enthesitis pathogenesis, but also function as a potential biomarker of enthesitis in PsA. These results should be taken with caution given previously described associations between PASI, BMI, and enthesitis [29, 30], the effect of the treatment, and the difficulty to score enthesitis in PsO patients, in which subclinical enthesitis is quite common [29, 31, 32]. This could explain why the associations between enthesitis and oxylipins were more robust in PsA patients. However, in our study, there was a trend towards an inverse relation between PASI and enthesitis in PsA patients, although it was not statistically significant (*r*^2^ = − 0.427, *p* = 0.674), and we did not observe any association between BMI and oxylipins in the PsO group.

Since oxylipins associated with enthesitis belong to COX2 and LOX pathways, dual inhibition could be beneficial in patients with enthesitis. Of note, even though TNF alpha inhibition decreased COX2 upregulation and PGE2 production in vitro, downregulation of COX2 expression after TNF treatment was not observed in ex vivo studies on synovial tissue from RA patients [22, 33]. Other studies in RA suggested that COX and LOX pathways remain overexpressed and can contribute to subclinical inflammation and relapse of rheumatic diseases [20]. Dual inhibition was explored in drug development. Yet, although these drugs were effective and had a better risk profile regarding cardiovascular and gastrointestinal damage compared to NSAIDs, they never reached the clinical stage mostly due to their liver toxicity [34]. In addition, evidence from animal models shows that 12/15 LOX, the murine enzyme equivalent to the human 15-LOX, has a protective role in inflammatory arthritis [35], and some LOX-derived oxylipins were shown to have anti-inflammatory properties [36, 37]. A better understanding of oxylipin pathways is needed to help discover new therapeutic strategies.

PLS-DA revealed an overlap of the oxylipin profiles between the 2 diseases. This finding is not surprising, since PsA patients also had skin involvement. A previous study that compared the lipidomic profile in lymphocytes from patients with PsO, with PsA, and healthy controls also found that the profiles of PsA and PsO overlapped, although they were clearly different from healthy controls [38]. This overlap explains the difficulty of validating prior results when using such a heterogenous population, despite the statistical methods used to adjust for confounding factors.

## Conclusions

This study and others suggest that an imbalance in oxylipin production is involved in the pathogenesis of psoriatic disease and our results provide a foundation for more focused investigations into psoriatic disease, and specifically in enthesitis pathogenesis and in novel eicosanoid-based interventions. Although our findings are promising, this study is not without its limitations. It does not include a control group of individuals without psoriatic disease, or patients with early disease without treatment, and the size of the cohort is small, which limits the generalization of our findings in a disease with a big overlap in its clinical manifestations. Additionally, LEI was used for the assessment of enthesitis, which only includes 6 entheseal sites. For future studies, we will consider the use of more comprehensive scores, such as SPARCC. The use of non-fasting samples is another limitation of our study, since diet can affect the concentrations of both the oxylipins and their precursors. The patients included in the study were from the same geographical area and probably had similar dietary patterns. Moreover, we did not detect any outlier concentrations, which would be expected if a patient had a very different dietary ingestion. It would also be of interest to study the distribution of oxylipins in the inflamed synovial tissue and to evaluate the relation between local and circulating oxylipins.

## Supplementary Information

**Additional file 1: Supplementary Figure 1.** Oxylipin synthesis pathways. Oxylipin synthesis from n3 and n6 precursors and the enzymes involved in their synthesis are shown. Pro-inflammatory oxylipins are marked in red, while anti-inflammatory ones are marked in blue. The precursor n3-PUFAs are marked in blue, while the n6-PUFAs are marked in red. **Supplementary Figure 2.** Spearman correlation between oxylipins concentrations in A) PsA and B) PsO patients. **Supplementary Figure 3.** Pro- and anti-inflammatory eicosanoids associated with BSA. Logistic regression was performed between each eicosanoid (pmol/ml) in patients with BSA ≤ 3 compared with patients with BSA > 3. A) Pro-inflammatory eicosanoids with p value < 0.1 after adjusting for BMI, DAS28-CRP, NSAIDs and biological therapy. B) Anti-inflammatory eicosanoids with p value < 0.1 after adjusting for BMI, DAS28-CRP, NSAIDs and biological therapy. Other factors, including comorbidities, gender and age were not found to influence eicosanoid levels and were not included in the model. C) Significant eicosanoids (p < 0.1) are circled in green if upregulated in patients with BSA > 3, and in red if downregulated in these patients. BMI body mass index, DAS28-CRP: disease activity score using the 28 joint count and C reactive protein; NSAIDs: non-steroidal anti-inflammatory drugs.

**Additional file 2: Supplementary Table 1.** Characteristics of PsO patients with low (PASI ≤ 2.5) and high (PASI > 2.5) skin disease activity. **Supplementary Table 2.** Comparison of oxylipins between PsO patients with low (PASI ≤ 2.5) and high (PASI > 2.5) skin disease activity. The average value for each oxylipin is presented, along with the significance of the comparison. Significantly different oxylipins are marked in red. **Supplementary Table 3.** Correlation of oxylipins with PASI in PsO patients. The correlation coefficients for each oxylipin are presented, along with the significance level. Significant correlations are marked in red. **Supplementary Table 4.** Correlation of oxylipins with BMI in PsA patients. The correlation coefficients for each oxylipin are presented, along with the significance level. Significant correlations are marked in red. **Supplementary Table 5.** Characteristics of PsA patients with low (PASI ≤ 2.5) and high (PASI > 2.5) skin disease activity. **Supplementary Table 6.** Comparison of oxylipins in PsA patients with high (PASI > 2.5) versus low (PASI ≤ 2.5) skin disease activity. The average value for each oxylipin is presented, along with the significance of the comparison. Significantly different oxylipins are marked in red. **Supplementary Table 7.** Correlation of oxylipins with PASI in PsA patients. The correlation coefficients for each oxylipin are presented, along with the significance level. Significant correlations are marked in red. **Supplementary Table 8.** Comparison of oxylipins in patients with high (PASI > 2.5) versus low (PASI ≤ 2.5) skin disease activity (both cohorts combined). The average value for each oxylipin is presented, along with the significance of the comparison. Significantly different oxylipins are marked in red. **Supplementary Table 9.** Correlation of oxylipins with mNAPSI (both cohorts combined). The correlation coefficients for each oxylipin are presented, along with the significance level. Significant correlations are marked in red. **Supplementary Table 10.** Comparison of oxylipins between patients with cDAPSA < 13 and patients with cDAPSA > 13. The average value for each oxylipin is presented, along with the significance of the comparison. Significantly different oxylipins are marked in red. **Supplementary Table 11.** Comparison of oxylipins between PsA patients with cDAPSA < 27 and patients with cDAPSA < 27. The average value for each oxylipin is presented, along with the significance of the comparison. Significantly different oxylipins are marked in red. **Supplementary Table 12.** Characteristics of PsA patients with enthesitis versus PsA patients without enthesitis. Supplementary Table 13. Comparison of oxylipins in PsA patients with enthesitis versus PsA patients without enthesitis. The average value for each oxylipin is presented, along with the significance of the comparison. Significantly different oxylipins are marked in red. **Supplementary Table 14.** Comparison of oxylipins in PsO patients with enthesitis versus PsO patients without enthesitis. The average value for each oxylipin is presented, along with the significance of the comparison. Significantly different oxylipins are marked in red. **Supplementary Table 15.** Comparison of oxylipins in patients with enthesitis versus patients without enthesitis (both cohorts combined). The average value for each oxylipin is presented, along with the significance of the comparison. Significantly different oxylipins are marked in red. **Supplementary Table 16.** Characteristics of PsO patients with enthesitis versus PsA patients without enthesitis. **Supplementary Table 17.** Comparison of oxylipins in PsO versus PsA patients. The average value for each oxylipin is presented, along with the significance of the comparison. Significantly different oxylipins are marked in red.

## Data Availability

All data generated or analyzed during this study are included in this published article and its supplementary information files.

## Abbreviations

PsA: Psoriatic arthritis
PsO: Psoriasis
VAS: Visual analog scale
TJC: Tender joint count
SJC: Swollen joint count
cDAPSA: Clinical Disease Activity Index for Psoriatic Arthritis
PASI: Psoriasis Area and Severity Index
BSA: Body surface area
BMI: Body mass index
AA: Arachidonic acid
COX: Cyclooxygenase
LOX: Lipoxygenase
PUFA: Polyunsaturated fatty acids
DHA: Docosahexaenoic acid
EPA: Eicosapentaenoic acid
DGLA: Dihomo-gamma linolenic acid
LA: Linoleic acid
LC-MS: Liquid chromatography coupled with mass spectrometry
UPLC: Ultra-performance liquid chromatography
MRM: Multiple reaction monitoring
PLS-DA: Partial Least Square Regression Discriminant Analysis
DMARD: Disease-modifying anti-rheumatic drug
TNF: Tumor necrosis factor
NSAID: Non-steroid anti-inflammatory drug
NMR: Nuclear magnetic resonance
Th: T helper
IL: Interleukin
DAS28: Disease activity score
VIP: Variable importance in projection
