# Supplementary material for: Differences in oxylipin profile in psoriasis versus psoriatic arthritis
Source: Arthritis Res Ther. 2021 Jul 24;23:200. doi: 10.1186/s13075-021-02575-y (PMC8310583; doi:10.1186/s13075-021-02575-y)
Supplement: Supplementary file 1 — Additional file 1: Supplementary Figure 1. Oxylipin synthesis pathways. Oxylipin synthesis from n3 and n6 precursors and the enzymes involved in their synthesis are shown. Pro-inflammatory oxylipins are marked in red, while anti-inflammatory ones are marked in blue. The precursor n3-PUFAs are marked in blue, while the n6-PUFAs are marked in red. Supplementary Figure 2. Spearman correlation between oxylipins concentrations in A) PsA and B) PsO patients. Supplementary Figure 3. Pro- and anti-inflammatory eicosanoids associated with BSA. Logistic regression was performed between each eicosanoid (pmol/ml) in patients with BSA ≤ 3 compared with patients with BSA > 3. A) Pro-inflammatory eicosanoids with p value < 0.1 after adjusting for BMI, DAS28-CRP, NSAIDs and biological therapy. B) Anti-inflammatory eicosanoids with p value < 0.1 after adjusting for BMI, DAS28-CRP, NSAIDs and biological therapy. Other factors, including comorbidities, gender and age were not found to influence eicosanoid levels and were not included in the model. C) Significant eicosanoids (p < 0.1) are circled in green if upregulated in patients with BSA > 3, and in red if downregulated in these patients. BMI body mass index, DAS28-CRP: disease activity score using the 28 joint count and C reactive protein; NSAIDs: non-steroidal anti-inflammatory drugs. [file 13075_2021_2575_MOESM1_ESM.pptx]

## Slide 1
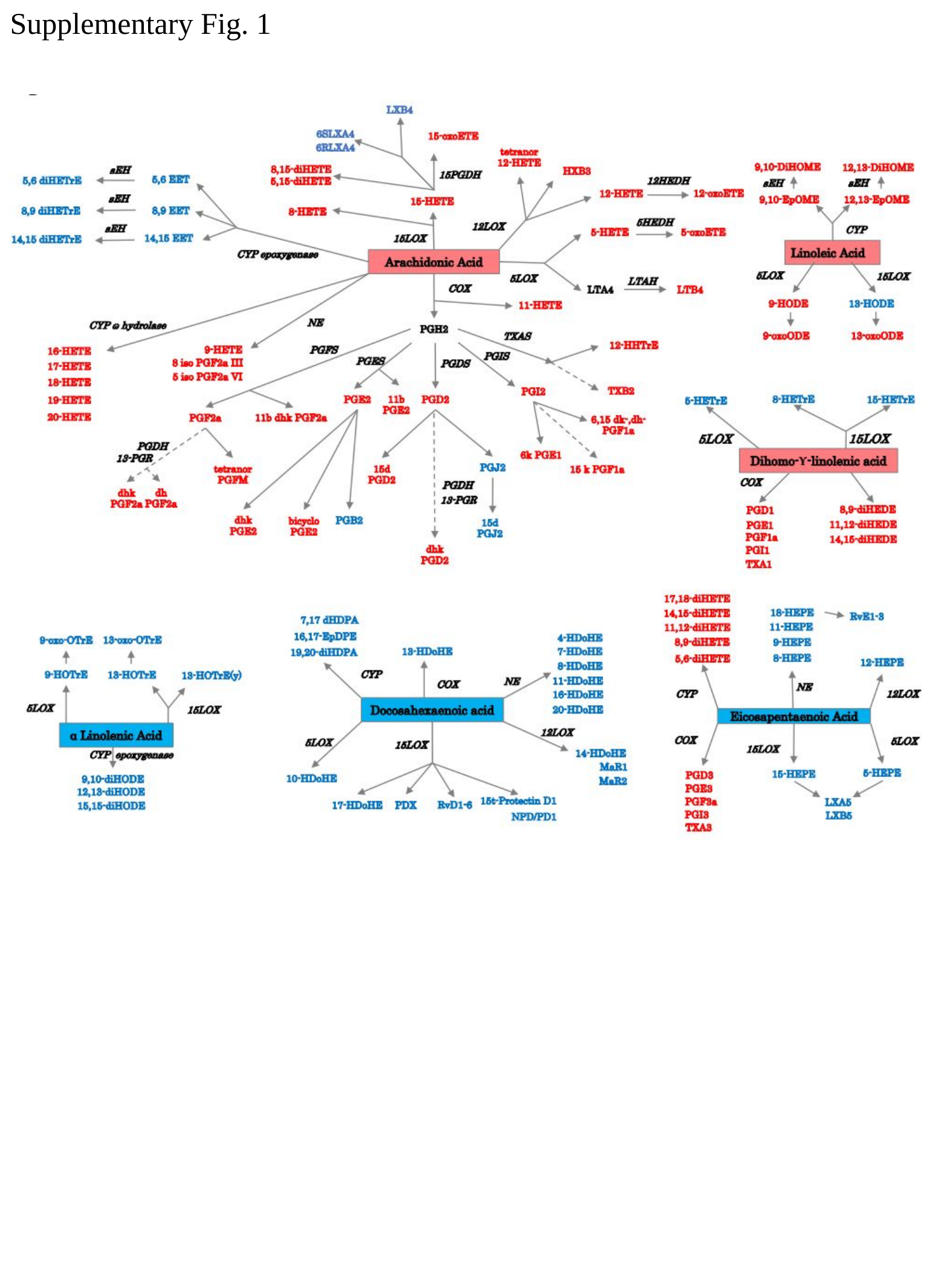

Supplementary Fig. 1

## Slide 2
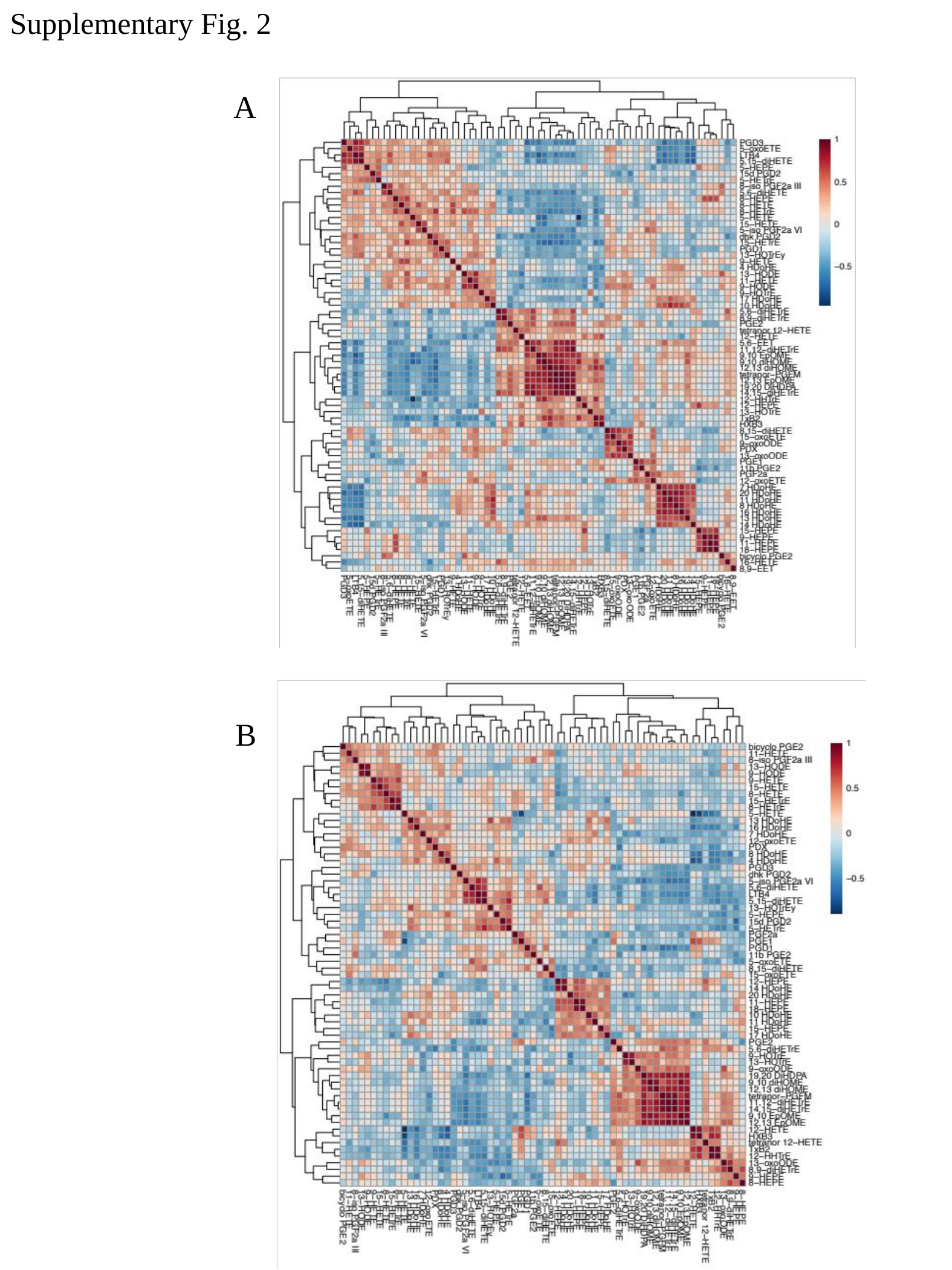

Supplementary Fig. 2
A
B

## Slide 3
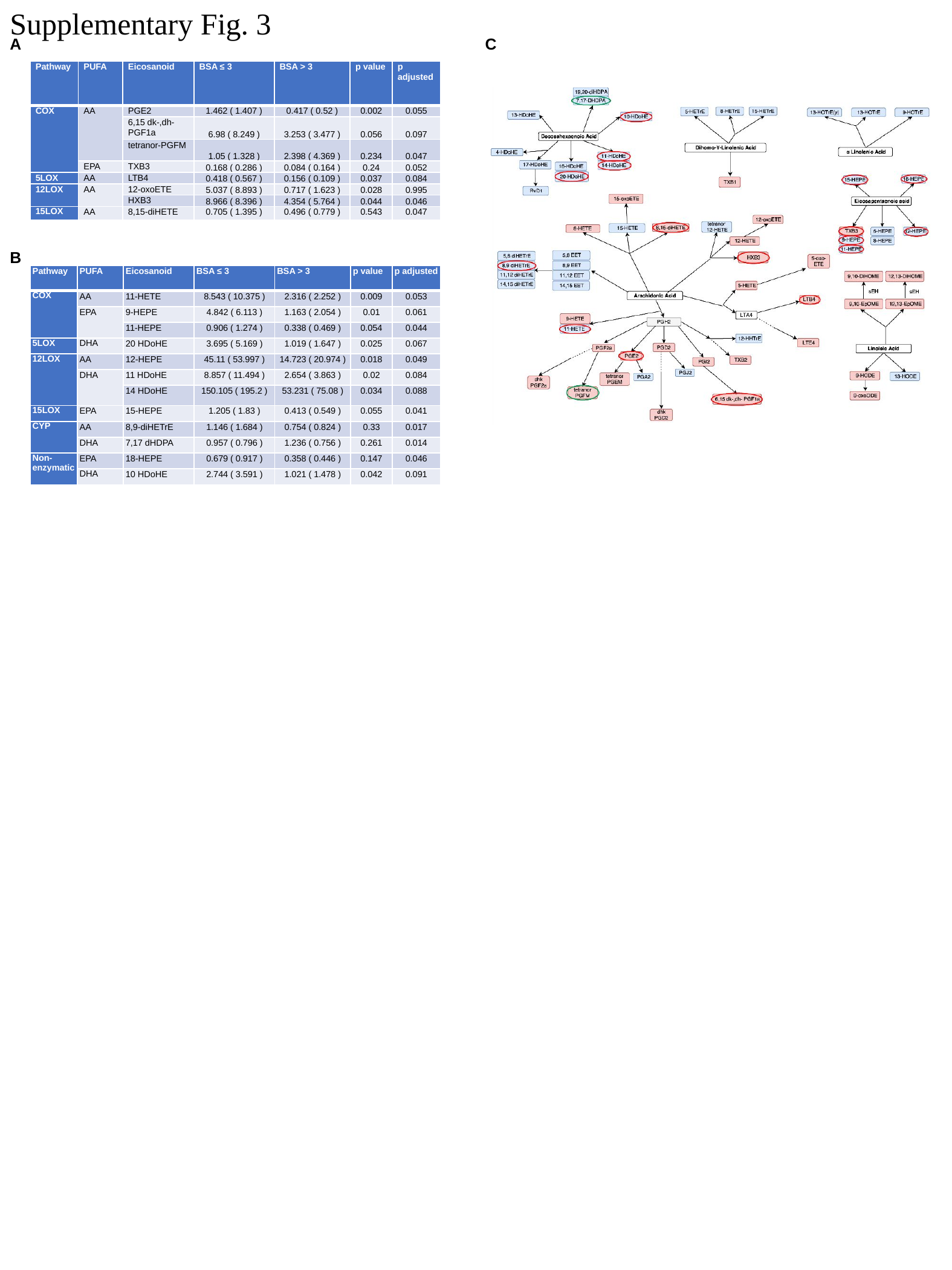

Supplementary Fig. 3
A
C
| Pathway | PUFA | Eicosanoid | BSA ≤ 3 | BSA > 3 | p value | p adjusted |
| --- | --- | --- | --- | --- | --- | --- |
| COX | AA | PGE2 | 1.462 ( 1.407 ) | 0.417 ( 0.52 ) | 0.002 | 0.055 |
| | | 6,15 dk-,dh- PGF1a | 6.98 ( 8.249 ) | 3.253 ( 3.477 ) | 0.056 | 0.097 |
| | | tetranor-PGFM | 1.05 ( 1.328 ) | 2.398 ( 4.369 ) | 0.234 | 0.047 |
| | EPA | TXB3 | 0.168 ( 0.286 ) | 0.084 ( 0.164 ) | 0.24 | 0.052 |
| 5LOX | AA | LTB4 | 0.418 ( 0.567 ) | 0.156 ( 0.109 ) | 0.037 | 0.084 |
| 12LOX | AA | 12-oxoETE | 5.037 ( 8.893 ) | 0.717 ( 1.623 ) | 0.028 | 0.995 |
| | | HXB3 | 8.966 ( 8.396 ) | 4.354 ( 5.764 ) | 0.044 | 0.046 |
| 15LOX | AA | 8,15-diHETE | 0.705 ( 1.395 ) | 0.496 ( 0.779 ) | 0.543 | 0.047 |
B
| Pathway | PUFA | Eicosanoid | BSA ≤ 3 | BSA > 3 | p value | p adjusted |
| --- | --- | --- | --- | --- | --- | --- |
| COX | AA | 11-HETE | 8.543 ( 10.375 ) | 2.316 ( 2.252 ) | 0.009 | 0.053 |
| | EPA | 9-HEPE | 4.842 ( 6.113 ) | 1.163 ( 2.054 ) | 0.01 | 0.061 |
| | | 11-HEPE | 0.906 ( 1.274 ) | 0.338 ( 0.469 ) | 0.054 | 0.044 |
| 5LOX | DHA | 20 HDoHE | 3.695 ( 5.169 ) | 1.019 ( 1.647 ) | 0.025 | 0.067 |
| 12LOX | AA | 12-HEPE | 45.11 ( 53.997 ) | 14.723 ( 20.974 ) | 0.018 | 0.049 |
| | DHA | 11 HDoHE | 8.857 ( 11.494 ) | 2.654 ( 3.863 ) | 0.02 | 0.084 |
| | | 14 HDoHE | 150.105 ( 195.2 ) | 53.231 ( 75.08 ) | 0.034 | 0.088 |
| 15LOX | EPA | 15-HEPE | 1.205 ( 1.83 ) | 0.413 ( 0.549 ) | 0.055 | 0.041 |
| CYP | AA | 8,9-diHETrE | 1.146 ( 1.684 ) | 0.754 ( 0.824 ) | 0.33 | 0.017 |
| | DHA | 7,17 dHDPA | 0.957 ( 0.796 ) | 1.236 ( 0.756 ) | 0.261 | 0.014 |
| Non-enzymatic | EPA | 18-HEPE | 0.679 ( 0.917 ) | 0.358 ( 0.446 ) | 0.147 | 0.046 |
| | DHA | 10 HDoHE | 2.744 ( 3.591 ) | 1.021 ( 1.478 ) | 0.042 | 0.091 |
